# Plant Peroxisomal Polyamine Oxidase: A Ubiquitous Enzyme Involved in Abiotic Stress Tolerance

**DOI:** 10.3390/plants12030652

**Published:** 2023-02-01

**Authors:** Ishita Samanta, Pamela Chanda Roy, Eshani Das, Sasmita Mishra, Gopal Chowdhary

**Affiliations:** 1 Plant Molecular Biology Laboratory, School of Biotechnology, KIIT, Bhubaneswar 751024, India; 2 Department of Biology, Kean University, 1000 Morris Avenue, Union, NJ 07083, USA

**Keywords:** polyamine oxidase, abiotic stress, peroxisome, three-dimensional structure, evolution

## Abstract

Polyamines (PAs) are positively charged amines that are present in all organisms. In addition to their functions specific to growth and development, they are involved in responding to various biotic and abiotic stress tolerance functions. The appropriate concentration of PA in the cell is maintained by a delicate balance between the catabolism and anabolism of PAs, which is primarily driven by two enzymes, namely diamine oxidase and polyamine oxidase (PAO). PAOs have been found to be localized in multiple subcellular locations, including peroxisomes. This paper presents a holistic account of peroxisomal PAOs. PAOs are flavin adenine dinucleotide-dependent enzymes with varying degrees of substrate specificity. They are expressed differentially upon various abiotic stress conditions, namely heat, cold, salinity, and dehydration. It has also been observed that in a particular species, the various PAO isoforms are expressed differentially with a spatial and temporal distinction. PAOs are targeted to peroxisome via a peroxisomal targeting signal (PTS) type 1. We conducted an extensive bioinformatics analysis of PTS1s present in various peroxisomal PAOs and present a consensus peroxisome targeting signal present in PAOs. Furthermore, we also propose an evolutionary perspective of peroxisomal PAOs. PAOs localized in plant peroxisomes are of potential importance in abiotic stress tolerance since peroxisomes are one of the nodal centers of reactive oxygen species (ROS) homeostasis and an increase in ROS is a major indicator of the plant being in stress conditions; hence, in the future, PAO enzymes could be used as a key candidate for generating abiotic stress tolerant crops.

## 1. Introduction

Polyamines (PAs) are small aliphatic amines with four to ten carbon chain lengths and are ubiquitously present across the kingdoms, from prokaryotic to eukaryotic organisms. Polyamines may exist in multiple forms, such as free-polyamines, covalently conjugated, or non-covalently conjugated (NCC-PAs) forms [1]. The largest pool of PAs is constituted by free polyamines conjugated with phenolic compounds, such as hydroxycinnamic acid, coumaric acid, caffeic acid, or ferulic acid via amide linkage [2,3,4]. In the physiological state, the free PAs exist as fully protonated and positively charged, and hence make a complex with macromolecules, such as nucleic acids, proteins, or lignin via ionic interaction or hydrogen bonding [5]. The most common polyamines in higher plants are putrescine (Put, a di-amine), spermidine (Spd, a tri-amine), and spermine (Spm, a tetra-amine) [3,6,7]. In contrast, in lower plants, like algae and mosses, the unusual PAs, namely norspermidine (NorSpd) and norspermine (NorSpm), constitute the bulk of PAs [8]. Structurally, NorSpd and NorSpm are similar to their more commonly present PA siblings Spd and Spm respectively, except they have one methyl group less in the carbon chain [9,10]. Lately, these unusual polyamines have also been identified in low concentrations in higher plants like *Medicago sativa* [8,11,12], *Arabidopsis thaliana* [13], and *Zea mays* [14]. Another tetra-amine, thermospermine (T-Spm), has been identified both in the lower plant, a diatom (*Thalassiosira pseudonana*), and in the higher plant *A. thaliana* [13]. Another secondary diamine similar to Put is cadaverine, which has been reported in *Glycine max* seedlings [15]. Studies pertaining to PAs such as T-Spm, NorSpd, NorSpm, and cadaverine are still scarce in the literature.

Traditionally, there are three major PAs present in plants: Put, Spm and Spd. However, with more and more data being made available, a fourth PA, namely T-Spm, has also been added to this category. All the PAs have been implicated to have both some common and specific functions. In plants, PAs have been suggested to be involved in a wide range of functions, starting from embryogenesis to flowering and senescence [6,7,16]. Their role in biotic and abiotic stress tolerance has also been documented [3,16,17]. T-Spm, which has been demonstrated to be required for stem elongation in *A. thaliana*, has also been considered a major PA in higher plants [14,18], while the secondary diamine cadaverine has been reported to be required for root growth in *G*. *max* seedlings [15].

PA concentrations in cells fluctuate, and they are governed by a dynamic balance of anabolism and catabolism. Polyamine oxidases (PAOs) play a significant role in PA metabolism and they are therefore of much importance in maintaining the cellular pool of polyamines. PAOs are primarily present in the cytosol and apoplast; however, lately, their peroxisomal localization has also been reported in various plant species. Peroxisomes are an important organelle for abiotic stress responses. Hence in this review, we present a detailed account of plant PAOs with special reference to peroxisomal PAOs.

## 2. Polyamine Metabolism

### 2.1. Catabolism

The two important enzymes involved in polyamine catabolism are diamine oxidase (DAO) and polyamine oxidase (PAO) [4,19,20]. DAO uses Cu^2+^ and pyridoxal phosphate as cofactors. It acts upon Put and converts it to 4-aminobutanal with concomitant production of H_2_O_2_ and NH_3_. The 4-aminobutanal is acted upon by the pyrrolinedehydrogenase (PYRR-DH) enzyme and is converted to γ-aminobutyric acid (GABA), followed by conversion to Krebs cycle intermediate, succinate. In comparison to monocots, dicot plants contain higher amounts of DAOs; however, their encoding genes have been cloned from very few plant species [20].

**Figure 1:**
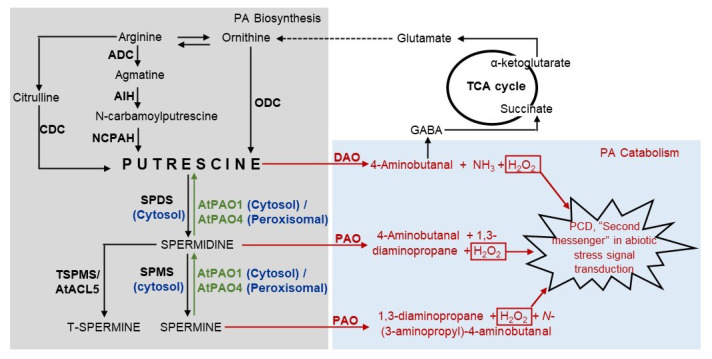
Diagrammatic representation of PA catabolism, anabolism, and their links with the TCA cycle. Grey and blue highlighted boxes represent biosynthesis and catabolism, respectively. Enzymes are represented by bold text. Green texts and arrows represent back conversions. Red texts and arrows represent catabolism. Dotted arrow represents the multi-step conversion. ACL—ACAULIS, ADC—arginine decarboxylase, AIH—agmatine iminohydrolase, CDC—citrulline decarboxylase, DAO—diamine oxidase, GABA—γ-aminobutyric acid, NCPAH—N-carbamoylputrescine amidohydrolase, ODC—ornithine decarboxylase, PAO—polyamine oxidase, PCD—programmed cell death, SPDS—spermidine synthase, SPMS—spermine synthase, TCA—tricarboxylic acid, TSPMS—thermospermine synthase.

PAO is a flavin adenine dinucleotide (FAD)-dependent enzyme and catalyzes the oxidative deamination of PAs at both the secondary amino groups [19,20]. Unlike DAOs, they have been found to remain present in monocots at high levels [21,22]. PAO enzymes are of two types: terminal catabolism (TC) and back conversion (BC) type. TC type leads to the breakdown of PAs into corresponding aldehydes: 4-aminobutanal and *N*-(3-aminopropyl)-4-aminobutanal, for Spd and Spm, respectively, along with concomitant production of 1,3-diaminopropane and hydrogen peroxide (H_2_O_2_), while the BC-type PAO leads to conversion of tetramine to triamine, and in certain circumstances, of triamine to diamine, leading to an increase in the cellular concentration of PAs [19,23,24,25,26]. The H_2_O_2_, which is produced as a byproduct of PA catabolism, has been demonstrated to act as a second messenger in biotic and abiotic stress signal transduction pathways [27,28] (Figure 1). It also affects the closure of stomata mediated by abscisic acid [20,29,30]. It has also been speculated that PAs lead to the accumulation of another second messenger, nitric oxide [31], which has also been deemed necessary for plant growth and development aside from its involvement in biotic and abiotic stress signaling [32].

### 2.2. Biosynthesis

The diamine, Put, is the central compound of PA biosynthesis. In plants, Put is synthesized from two different precursors—ornithine and arginine. Ornithine is converted to Put by the enzyme ornithine decarboxylase (ODC) in a single-step reaction [33,34]. Arginine is converted to Put in a three-step enzymatic reaction, where arginine decarboxylase (ADC) converts arginine to agmatine and carbon dioxide. In the second step, agmatine is converted to N-carbamoylputrescine (NCPA) and ammonia by the enzyme agmatine iminohydrolase (AIH). In the last step the N-carbamolylputrescine amidohydrolase (NCPAH) hydrolyses N-carbamoylputrescine to Put, CO_2_ and NH_3_ [34]. This is the primary Put biosynthesis pathway in plants [35,36]. There lies another alternate pathway, where arginine is converted to Put via an intermediate, citrulline, by the enzyme citrulline decarboxylase (CDC) [37,38,39]. The biosynthesis of Put via citrulline is limited in occurrence and has been reported in *Sesamum indicum* plants only [40]. It has also been observed that the gene ODC has been lost from *A. thaliana* and many other members of Brassicaceae during the course of evolution [41], suggesting that the ODC-dependent pathway may not be absolutely necessary for normal growth and development [40]. The diamine Put is converted to triamine Spd by the enzyme spermidine synthase, which has been found to be localized in cytosolic fractions [42]. The latter is further converted into tetra-amines Spm and T-Spm by spermine synthase (SPMS) and thermospermine synthase (T-SPMS), respectively, reviewed in [33]. These enzymes catalyze the addition of a fourth amine group. In *A. thaliana*, ACAULIS5 (ACL5) has been demonstrated to be a thermospermine synthase ortholog, which synthesizes T-Spm from Spd [13,14,43] (Figure 1).

The BC-type PAOs also contribute to the accumulation of the cellular PA pool. The recombinant AtPAO1 has been found to catalyze the back conversion of tetramine Spm and NorSpm to triamine Spd and NorSpd, respectively [44]. In the case of rice, all the three peroxisomal PAOs (OsPAO3, OsPAO4, and OsPAO5) and one cytosolic isoform OsPAO1, carry out PA back conversion from Spm and T-Spm to Spd and Spd to Put [19,45]. The BC-type PAO has also been reported in the lower plant, *Selaginella lepidophylla*, where it (SelPAO) catalyzes the back conversion of Spm and T-Spm to Spd and NorSpd, respectively. Usually, NorSpd is synthesized from 1,3-diaminopropane (DAP) by the action of aminopropyl transferase (APT). SelPAO synthesis of NorSpd from T-Spm reveals a novel pathway for NorSpd synthesis [8]. From the back-conversion property of the PAO, it may be envisioned that PAO enzymes play a crucial role in maintaining the cellular concentration of polyamines as they are involved both in the catabolism and anabolism of PA, thereby regulating the PAO enzymes, which could be instrumental in the polyamine-dependent stress adaption of plants.

## 3. Polyamine Oxidases

### 3.1. Substrate Specificity

#### 3.1.1. Dicot PAOs

**Figure 2:**
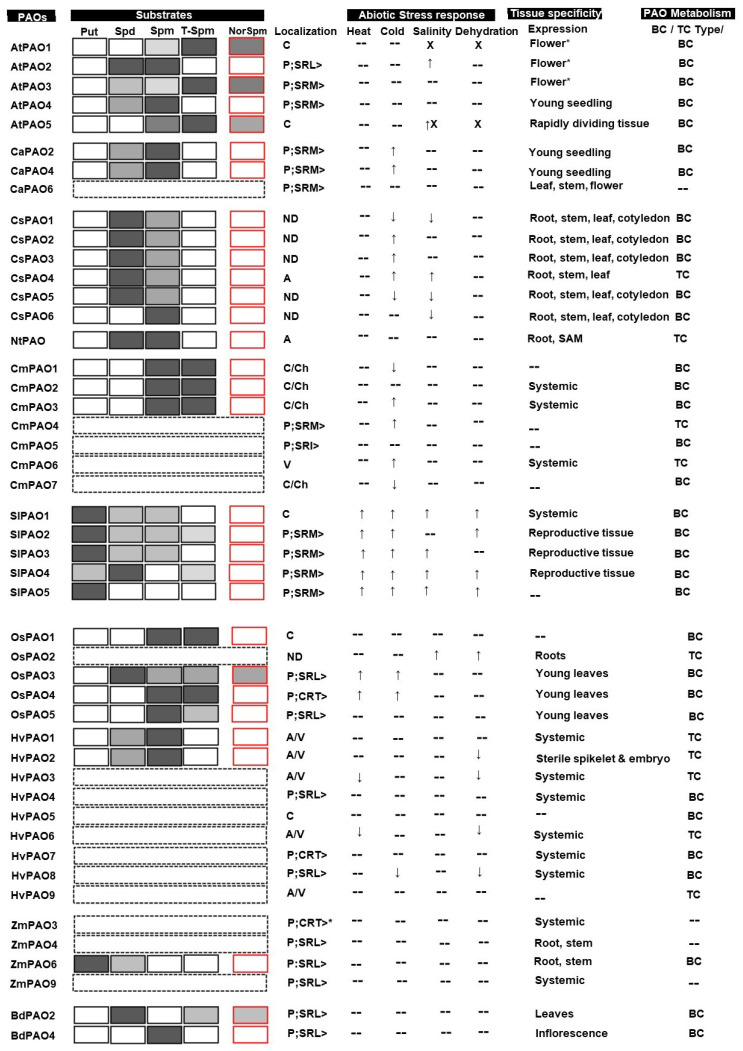
Diagrammatic representation of various properties of PAO enzymes. The left column shows the PAO enzymes from various plant species. The substrates of PAO enzymes are shown with rectangular boxes. The preference of substrates by the respective PAO enzyme is shown with color gradation. The most preferred substrate has the densest color. The PA substrate that is not used by the respective PAO is left blank. The unusual PA (NorSpm) rectangles are bordered with red, while the usual PA (Put, Spd, Spm, and T-Spm) rectangles are bordered with black. The PAO enzyme for which no substrate utilization information is known is shown with a dashed-bordered rectangle. The subcellular localization of the respective PAO is represented next to the substrate column. C—cytoplasm; P—peroxisome, which is followed by three amino acids representing the PTS1; “>” represents the end of the polypeptide chain; A—Apoplast; V—Vacuole; Ch—Chloroplast; ND—not determined; SAM—shoot apical meristem; “*”—also expressed in other parts of the plant, but the highest level of expression is observed in the flower. Following the localization column, lies the abiotic expression induction columns. “↑”—upregulation; “↓”—downregulation; “X”—mutation studies leading to stress tolerance; “--” no studies. The extreme right column shows the metabolism type of respective PAO; BC—back conversion-type; TC—terminal catabolism-type. The PAOs for which no data is available (CaPAO1, CaPAO3, CaPAO5, ZmPAO1, ZmPAO2, ZmPAO5, ZmPAO7, ZmPAO8) are not shown.

As per the recombinant PAO-dependent enzyme assays, the substrate specificity of various PAO enzymes is mostly restricted to Spm, Spd, and rarely, Put [16,46]. Figure 2 provides a pictographic summary of the substrate specificity of various PAO enzymes. Recombinant AtPAO1, produced in *E. coli* as hexahistidine-tagged enzymes, only oxidizes Spm and not Spd [9,44], while the preferred substrate of AtPAO3 is Spd [9,47], as deduced from the *k_cat_*/*K_m_* values where Spd is twice the preferable Spm. AtPAO2 and AtPAO4 oxidize both Spm and Spd but do not act on Put, however; AtPAO2 acts upon both the substrates with equal affinity, as is exhibited by their similar *k_cat_*/*K_m_* values, while AtPAO4 has 40 times more affinity towards Spm than Spd, as determined by their *k_cat_*/*K_m_* values. It has also been observed that AtPAO1 prefers T-Spm over Spm [44], which suggests this may be its physiological substrate. Recombinant AtPAO2 and AtPAO4 have also been found to oxidize the artificial substrate, *N*^1^-acetyl-spermine; however, with a much lower efficiency of 3.9 and 3.4 times, respectively, when compared to their preferred substrates [9]. The preferred substrate of AtPAO5 is T-Spm, followed by NorSpm and Spm. This has also been found to act upon the artificial substrate *N*^1^-acetyl-spermine with comparatively lower affinity compared to T-Spm [45].

In the case of *Capsicum annuum*, CaPAO2 and CaPAO4 prefer Spm over Spd [48], while in the case of *Citrus sinensis*, all the PAOs, namely CsPAO1, CsPAO2, CsPAO3, CsPAO4, and CsPAO5, prefer Spd over Spm, except CsPAO6, which only acts upon Spm [49]. The PAO from *Nicotiana tabacum* (NtPAO) acts upon both Spd and Spm with equal affinity [17]. *Camellia sinensis* PAO1, CmPAO2, and CmPAO3 (in the literature, the *Camellia sinensis* PAOs are abbreviated as CsPAO; however, in this paper to distinguish them from *Citrus sinensis* PAOs, we have abbreviated them as CmPAO) prefers Spm as their usual substrate [50]. None of the PAOs reported are found to act upon Put, except *Solanum lycopersicum* PAO1, SlPAO2, SlPAO3, SlPAO4, and SlPAO5. SlPAO1-3 prefers Put over Spm/Spd, while SlPAO5 acts only on Put. Only SlPAO4 prefers Spm over Put [22].

Apart from the usual PAs, PAOs from higher plants have also been reported to act upon unusual PAs, such as NorSpm. Recombinant AtPAO1 shows 6 and 1.3 times more affinity towards T-Spm and NorSpm, respectively, when compared to its regular substrate Spm, suggesting that T-Spm and the unusual PA, NorSpm, may be its physiological substrate. Recombinant AtPAO3 also prefers NorSpm and T-Spm over Spd [44]. However, recombinant AtPAO2 and AtPAO4 proteins do not act upon NorSpm, suggesting that these PAOs are specific to standard PAs only. Moreover, the presence of T-Spm in *A*. *thaliana* plants has also been detected and found to be involved in various growth and developmental processes, such as vascular tissue formation. [51,52,53]. Furthermore, the exogenous application of T-Spm has been shown to rescue the *A*. *thaliana* plant from dwarf phenotype [18]. Apart from *A*. *thaliana*, *Camellia sinensis* PAO1, CmPAO2, and CmPAO3 act on T-Spm with equal affinity as with their usual substrate Spm [50]. SlPAO2 and SlPAO4 act upon T-Spm, but with reduced efficiency when as compared to their preferred substrates Put and Spm, respectively [22].

#### 3.1.2. Monocot PAOs

In the case of monocots, the substrate specificity has been determined using recombinant protein production, followed by enzyme assays. The PAO from *O*. *sativa*, namely OsPAO3, prefers Spd, followed by T-Spm and Spm, while OsPAO4 and OsPAO5 prefer Spm and T-Spm and act upon Spd. None of the OsPAOs act upon Put [19]. Recombinant *Hordeum vulgare* (Hv) PAO1 and HvPAO2 have been found to prefer Spm over Spd with 14-fold higher affinity [25]. ZmPAO6 is the only monocot PAO found to act on Put [54]. *Brachypodium distachyon* (Bd) PAO2 and BdPAO3 have been found to prefer Spd and Spm, respectively [21]. None of the monocot PAOs have either T-Spm or the unusual PA, NorSpm, as their preferred substrate. However, OsPAO1, OsPAO3, and OsPAO5 have been found to act on T-Spm, but with reduced affinity to their preferred substrate. OsPAO3 acts on NorSpm apart from T-Spm [55]. BdPAO2 acts on T-Spm as well as both the unusual PAs, NorSpm and NorSpd, but with reduced affinity with respect to its preferred substrate [21].

In the evolutionary lineage, once the plant life migrates to land, dicotyledons appear first, followed by monocotyledons. It has also been observed that lower plants contain unusual PAs as their primary cellular PA pool [55]. Hence, during the course of evolution, the dicots retained some of the PAO enzyme habits which prefer the unusual PAs over the usual PAs, as in the case of *A*. *thaliana*. Monocots that are further up in the phylogenetic lineage lose these PAO enzymatic properties, which prefer unusual PAs as their physiological substrate. However, some of the monocotyledonous PAOs act upon the unusual PA, such as OsPAO1, OsPAO3, OsPAO5, BdPAO2, etc., but with reduced affinity compared to the regular PAs. This further supports the idea that during evolution, PAO enzymes evolve towards using usual or regular PAs (Put, Spm, Spd, and T-Spm) as their substrates.

### 3.2. Tissue Specificity of PAOs

#### 3.2.1. Dicot PAOs

PAOs show a wide range of distribution in terms of their expression in various types of plant tissues. Figure 2 depicts a summary of the expression of PAOs in various tissue forms. Takahashi et al. [16] performed a detailed analysis of *A. thaliana* PAO gene expression studies. *AtPAO1* was highly expressed in flowers while almost undetectable levels of expression were found in young seedlings, rosette leaves, and stems. *AtPAO2* was found to be expressed in very low amounts in seedlings while its expression increased with the age of the plant and was abundantly expressed in the stem, and the highest level of expression was observed in the flower. *AtPAO3* was found to be constitutively expressed in all plant parts with the highest level of expression in flowers. *AtPAO4* was abundantly expressed in young seedlings and mature rosettes while its expression was minimal in stems and flowers, while *AtPAO5* was found to be expressed in rapidly dividing tissues [9,16,44,45]. In the case of *Citrus sinensis* and *Camellia sinensis*, the PAOs showed more systemic expression. *CsPAO1*, *CsPAO2*, *CsPAO3*, *CsPAO5*, and *CsPAO6* have been found to be expressed in near equal amounts in roots, stems, leaves, and cotyledon, while *CsPAO4* is highly expressed in roots, stems, and leaves, but not in cotyledon [48]. The PAOs from *Camellia sinensis*, namely *CmPAO2*, *CmPAO3*, and *CmPAO6* are expressed in all the tissues; however, the expression level of *CmPAO6* is relatively very low compared to *CmPAO2* and *CmPAO3* [50]. The PAO from *N*. *tabacum* (*NtPAO*)*,* is expressed only in shoot apical meristem and roots [17]. In the case of *C. annuum PAO2*, *CaPAO3* and *CaPAO4* are expressed in leaves and stems in the seedling stages. *CaPAO1* and *CaPAO6* are expressed in flowers, as well as in the leaves and stems of seedlings. The expression of *CaPAO6* also continues in mature leaves, while *CaPAO5* is constitutively expressed and present in all the tissues, although a very minimal level of expression is observed [48]. The PAOs from *S*. *lycopersicum* are primarily expressed in the reproductive parts, except for *SlPAO1* and *SlPAO7*, which are expressed in all vegetative tissues and roots, respectively; however, the level of expression of *SlPAO1* in the vegetative tissues is relatively low. *SlPAO2*, *SlPAO3*, and *SlPAO4* are expressed in reproductive tissues during another development only [22].

#### 3.2.2. Monocot PAOs

In the case of *O. sativa*, *OsPAO3, OsPAO4*, and *OsPAO5* are abundantly expressed in young leaves. *OsPAO2* is expressed in roots while the expression of *OsPAO1* and *OsPAO6* is barely detectable [19]. *OsPAO7* is exclusively expressed in the anthers [11]. The PAOs from *H. vulgare* (*HvPAO1*, *HvPAO3*, *HvPAO4*, *HvPAO6*, *HvPAO7*, and *HvPAO8*) are expressed systemically in all the vegetative tissues except *HvPAO2*, which is expressed in sterile spikelets and embryos [23,24]. Similarly, the PAOs from *T. aestivum* (*TaPAO3*, *TaPAO5*, *TaPAO8*, and *TaPAO11*) are expressed systemically in vegetative tissues; however, *TaPAO5*, *TaPAO8*, and *TaPAO11*, which are also found to be expressed in the reproductive parts [56]. The PAOs from *Z. mays* (*ZmPAO2*, *ZmPAO3*, *ZmPAO5*, *ZmPAO8*, and *ZmPAO9*) are systemically expressed in all the vegetative tissues, while *ZmPAO4* and *ZmPAO6* are expressed specifically in roots and stems only. The expression of *ZmPAO1* is restricted to seedlings only [54,57]. *BdPAO1* and *BdPAO2* from *B. distachyon* are found to be expressed in leaves, while *BdPAO3*, *BdPAO4*, and *BdPAO5* are found to be expressed in inflorescence only [21].

The presence of multiple PAO isoforms in a plant species, followed by their differential expression in different tissues under various developmental stages, suggests that PAO activity is essential to plant growth and development and the different isoforms may not be simply duplicating each other. This indicates that the isoforms may have specific and crucial functions at the specific developmental stage of the plant. However, in the future, mutation studies need to be performed to further confirm this.

### 3.3. Role of Polyamine Oxidases in Abiotic Stress Tolerances

Plants in nature are exposed to various kinds of biotic and abiotic stress and being sessile, they cannot escape the stress conditions but rather, they withstand it. The critical role of PAs in stress tolerance has been well documented. Elevations in the PA level have been found to impart stress tolerance in plants, reviewed in [58]. *AtPAO2* and *AtPAO5* are found to be upregulated by saline stress; however, *AtPAO1*, *AtPAO3*, and *AtPAO4* remain unaffected by salinity treatment [59]. Sagor et al. [60] generated a series of *A*. *thaliana* mutant lines where PAO genes were knocked out. An *atpao1* and *atpao5* double mutant for cytoplasmic PAOs was found to be tolerant to saline and dehydration stress, while another double mutant for peroxisomal PAOs, namely *atpao2*-*atpao4*, was found to be sensitive to saline and dehydration stress when compared with the wild type (WT). It was further observed that in the former double mutant line, PAO activity was reduced to 62% and the Na^+^ uptake was also reduced to 75% compared to the WT. The reactive oxygen species (ROS) production, which is considered a hallmark of abiotic stress, was also found to be reduced in the *atpao1*-*atpao5* double mutant lines. Furthermore, the level of T-Spm was also found to be higher in the double mutants compared to the WT. The saline and dehydration stress tolerance of cytoplasmic double mutant lines could be linked to the decline in the PAO catabolism by the AtPAO1 and AtPAO5 enzymes. This would ultimately lead to increased cellular PA concentration and reduced ROS production, thereby bringing about stress tolerance.

Furthermore, it has also been observed that the transcript levels of *A*. *thaliana* salt overly sensitive (*SOS*)*1*, *AtSOS2*, *AtSOS3*, and *NHX1* (a tonoplast Na^+^/H^+^ exchanger) [61] are higher in *atpao1*-*atpao5* double mutants. The *SOS* gene family has been deemed crucial for plant saline stress tolerance [62]. In addition to the SOS pathway, plants also respond to saline stress by inducing the abscisic acid (ABA) dependent and independent signaling pathways via various transcription factors (TF) [63]. In the case of the *atpao1*-*atpao5* double mutant, the transcript levels of ABA-dependent pathway TF genes *AREB1*, *AREB2*, and their target genes *RD29B* and *RAB18*, are found to be upregulated [60,64]. Similarly, the ABA-independent pathways TF genes *CBF3/DREB1A* and their target *RD29A* and *COR15A* have also been found to be upregulated in the *atpao1*-*atpao5* double mutants [60].

Apart from affecting the ABA-dependent and independent signaling pathways, the *atpao5* mutant has also been demonstrated to impart salinity tolerance to *A*. *thaliana* plants by influencing the jasmonic acid pathway. The *atpao5* single mutant line has been reported to have increased accumulation of jasmonic acid compared to the WT [59].

Similarly, the PAO from *C*. *annuum*, *CaPAO2*, and *CaPAO4* are upregulated upon cold stress and bring about freezing stress tolerance in *C*. *annuum*. When the *CaPAO2* and *CaPAO4* are overexpressed in *A*. *thaliana* plants, it leads to imparting freezing stress tolerance, which is mediated via upregulation of cold-responsive genes, namely *AtCOR15A*, *AtRD29A*, AtCOR47, and *AtKIN* [65]. The expression data from both *A*. *thaliana* and *C*. *annuum* suggests that PAO influences a battery of stress tolerance-related genes in plants. An independent study by Xiao et al. [48] reported a *CaPAO* isoform that was upregulated in response to saline and osmotic stress. PAO from *Citrus sinensis*, namely *CsPAO1* and *CsPAO5*, were found to be downregulated while *CsPAO2*, *CsPAO3*, and *CSPAO4* were found to be upregulated in response to cold stress. Similarly, in the case of saline stress, *CsPAO1*, *CsPAO5*, and *CsPAO6* were downregulated and *CsPAO4* was found to be upregulated [49]. The PAOs from *S*. *lycopersicum* also responded systemically to various abiotic stress conditions. Low and high-temperature stress led to the upregulation of *SlPAO1*, *SlPAO2*, *SlPAO3*, *SlPAO4*, *SlPAO5*, and *SlPAO6,* and *SlPAO1*, *SlPAO2*, *SlPAO3*, *SlPAO4*, and *SlPAO5*, respectively. Dehydration and salinity stress led to the upregulation of *SlPAO1*, *SlPAO2*, *SlPAO4*, *SlPAO5*, and *SlPAO1*, and *SlPAO3* and *SlPAO5*, respectively. *SlPAO6* and *SlPAO7* were found either to be downregulated (dehydration and salinity stress) or unaltered (low and high-temperature stress) [22]. The *O*. *sativa* PAOs were found to be responsive to heat, cold, salinity, and dehydration stress. *OsPAO3*, *OsPAO4*, and *OsPAO6* were upregulated in response to heat stress while cold stress led to the upregulation of *OsPAO4*, *OsPAO6*, and *OsPAO7*. The saline and dehydration stress leads to the upregulation of *OsPAO2* and *OsPAO6* [66]. Interestingly, the PAOs genes from *H*. *vulgare* are found to be only downregulated upon various abiotic stress conditions, namely heat (*HvPAO3* and *HvPAO6*), cold (*HvPAO8*), and dehydration (*HvPAO2*, *HvPAO3*, *HvPAO6*, and *HvPAO8*) [67] (Figure 2).

The expression pattern of multiple PAO genes with respect to various abiotic stress conditions has been documented; however, little is known about their signal transduction mechanism and the probable second messengers involved. PA metabolism by PAOs leads to the production of H_2_O_2_, whose role as a second messenger has also been established. It is believed that the PA-triggered salinity tolerance might be signaled through calcium as a second messenger [59,68], which modulates various ion channels [59,69], H^+^/ATPase activities [59,70], protein modification via S-nitrosylation, carbonylation, and tyrosine nitration [59] and ROS detoxification [59,71]. Furthermore, in *A*. *thaliana*, the absence of Spm accumulation leads to an imbalance in calcium homeostasis, which results in hypersensitivity to saline stress [68]. The export of PA to the apoplast region has been linked with the influx of calcium, leading to an increase in cytosolic calcium concentration [70]. The involvement of S-nitrosylation in the PA-mediated abiotic stress tolerance suggests that there could well be an indication of possible cross-talk between calcium and nitric oxide signaling. However, the direct involvement of calcium or nitric oxide as second messengers in the PAO-mediated abiotic stress signal transduction has not yet been demonstrated. It will be interesting to explore this direction in the future.

### 3.4. Three-Dimensional (3D) Structure of PAO

So far, the 3D structure of only one plant PAO has been resolved using x-ray crystallography, namely, ZmPAO [72]. The structure has been resolved with a resolution of 1.9 Å. ZmPAO, which is a monomeric 53-kDa protein containing 13 α-helices and 19 β-strands folding into two well-defined domains. The FAD binding domain comprises a central β sheet, which is flanked by a β strand and three α helices. The linear amino acid numbers 7–87, 187–292, and 412–466 constitute the FAD binding domain. The FAD binding domain is sunk deep in the structure. Five main amino acids, V237, E35, Y399, R43, and E430 play important roles in FAD binding.

**Figure 3:**
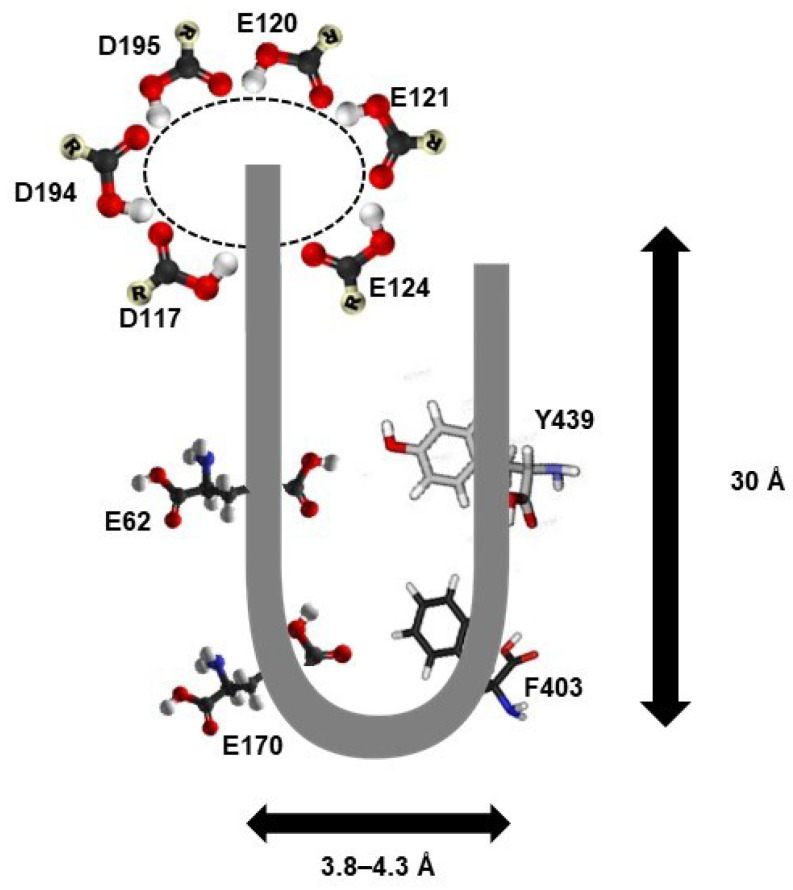
Diagrammatic representation of the active site of maize PAO. The PAO active site is represented by a characteristic U-shaped tunnel having a dimension of 30 × 3.8–4.3 Å. The side walls of the tunnel are lined by catalytic residues F403 and Y439, which make an aromatic sandwich. The base of the tunnel has E62 and E170. The mouth of the tunnel has an electron cloud created by E120, E121, E124, D117, D194, and D195. The solid lines represent the shape of the catalytic site. Red circle—oxygen, white—hydrogen, black—carbon, dashed circular line—electron cloud.

The substrate binding site is formed by a mixed six-stranded β-sheet flanked by five α-helices. This stretch is comprised of two domains (amino acids 88–186 and 293–411). The catalytic center of ZmPAO has a characteristic “U” shaped tunnel passing through the protein structure at the interface between the substrate and FAD binding domains. The U-shaped tunnel has a depth of 30 Å and an almost constant width of 3.8–4.3 Å. The opening of the “U” shaped catalytic site has a carboxylate ring framed by several solvent-accessible glutamate (E120, E121, and E124) and aspartate (D194 and D195) side chains. The sidechains of E62 and E170 protrude towards flavin, near the turning point of the tunnel. These two amino acids lie in close vicinity and can form H-bonds, suggesting that the E62–E170 pair is protonated. In the main body of the tunnel, F403 and Y439 are positioned parallel to each other and flank the tunnel on opposite sides making an aromatic sandwich (Figure 3). The 3D structure of ZmPAO describes the crucial amino acid residues present in the catalytic site and is responsible for PAO activity. This information could be used for understanding the catalytic center of newer PAOs whose 3D structures are yet to be deciphered.

The carboxylate ring made by aspartate and glutamate residues present at the entrance of the catalytic tunnel has been proposed to be the most crucial factor in binding the PAs (as substrate) into the catalytic tunnel [72]. The carboxylate ring being predominantly negatively charged attracts the positively charged PAs. The maize PAO has three aspartate and three glutamate side chains in the carboxylate rings. The number of amino acids present in the carboxylate ring may vary depending upon the PAO, which could be instrumental in deciding the substrate preference of PAO. A higher number of positive charges in the PA substrate may require a comparatively higher number of negatively charged amino acids in the carboxylate ring, in order to neutralize the charge and facilitate the interaction. However, to confirm this more three-dimensional PAO structure needs to be deciphered in the future.

### 3.5. Subcellular Localization of PAOs—Peroxisome Forms the Core of Intracellular PAOs

Traditionally PAOs are localized in the apoplastic region of plants. For instance, the N-terminal 22 amino acid of NtPAO constitutes a signal peptide for the secretory pathway with vesicle-mediated secretion into the apoplast, which has been demonstrated to be localized in apoplast via fluorescent protein tagging followed by fluorescent microscopy [17]. Of the intracellular sites, chloroplast, cytosol, and peroxisome are the sites where the localization of PAO has been demonstrated. While discussing subcellular localization in this paper, only experimentally verified ones were taken into account, and predictions were not considered (Figure 2).

*A. thaliana* contains five PAO isoforms, three of which, namely AtPAO2, AtPAO3, and AtPAO4, have been demonstrated to be localized in the peroxisome, while AtPAO1 and AtPAO5 are present in the cytosol [47,73]. Similarly, in the case of *S*. *lycopersicum*, out of the seven PAO isoforms, four PAOs, namely SlPAO2, SlPAO3, SlPAO4, and SlPAO5, have been demonstrated to be peroxisomal, while three PAOs, namely SlPAO1, SlPAO6, and SlPAO7, are found to be localized in the cytosol [22]. The two PAO isoforms from *C. annuum*, namely, CaPAO2 and CaPAO4, have been demonstrated to be peroxisomal; however, the localization of the rest of the CaPAOs has yet to be experimentally proven [65]. Another PAO from *C. annuum*, explained by Xiao et al. [48], has been demonstrated to be localized in the chloroplast. Similarly, in the case of *Citrus sinensis*, the subcellular localization of only CsPAO4 has been verified, and was found to be apoplast [49]. The PAOs from *Camellia sinensis* show the most diversity in terms of localization. CmPAO4 and CmPAO5 are peroxisomal, while CmPAO1, CmPAO2, CmPAO3, and CmPAO7 have been demonstrated to be dual localized in cytosol and chloroplast, while CsPAO6 is vacuolar [50]. *O*. *sativa* contains three peroxisomal PAOs, namely OsPAO3, OsPAO4, and OsPAO5, while OsPAO6 and OsPAO7 have been demonstrated to be apoplastic, and OsPAO1 is localized in the cytosol [19]. *H*. *vulgare* contains three peroxisomal PAOs, namely HvPAO4, HvPAO7, and HvPAO8, and five (HvPAO1, HvPAO2, HvPAO3, HvPAO6, and HvPAO9) dual-targeted (apoplast/vacuole). HvPAO5 is cytoplasmic in localization [23,24,25]. *Z*. *mays* also contains three peroxisomal PAO isoforms, namely ZmPAO4, ZmPAO6, and ZmPAO9 [57].

The peroxisomal targeting of PAOs is mediated by peroxisome targeting signal (PTS) type 1, which is located at the C-terminus of the protein and is primarily represented by the last three amino acids. Under certain circumstances, seven upstream residues also play a significant role in peroxisome targeting [74,75,76,77,78,79]. Since peroxisomes do not contain any genome of their own, all their proteome complement is encoded by the nuclear genome, synthesized on cytosolic ribosomes, and imported to peroxisomes via a signal-dependent manner [80]. The PTS1-containing proteins are recognized by cytosolic receptor peroxin (PEX) 5, which imports them to the peroxisomal matrix with the help of other PEX proteins [77,79,81]. As per the targeting efficiency, the PTS1 could be of canonical or non-canonical type. The canonical PTS1 leads to strong and efficient targeting, while the non-canonical PTS1 leads to the weak targeting of reporter proteins fused to PTS1-containing proteins [82]. The targeting efficiencies are based on in vitro studies. A typical canonical PTS1 is represented by [SA], [KR], and [LMI] > at −3, −2, and −1 positions, respectively. The PAO enzymes typically contain the canonical type of PTS1 and are represented primarily either by SRM> or SRL>. Exceptionally, one PAO isoform each from *Camellia sinensis* (CmPAO5), and *O*. *sativa* (OsPAO4) contains SRI> and CRT>, respectively [19,50]. Ono et al. [19] fused the OsPAO4 (containing CRT> as C-terminus tripeptide) with GFP and detected the green fluorescence in the peroxisomes. Cysteine at −3 position is a non-canonical residue and threonine at −1 position has not yet been demonstrated to be a functional residue in PTS1 [78]. Hence, we believe that CRT> may not be a functional PTS1; rather the protein is imported to the peroxisome via a piggy-backing mechanism. In the piggy-backing type of import mechanism, a protein that lacks the functional import signal binds to another protein containing the functional import signal and is imported to the specific subcellular site [83,84,85,86,87]. ZmPAO3 and HvPAO7 also contain CRT> as its C-terminus tripeptide, although it remains to be experimentally determined whether it is targeted to peroxisomes or not.

Peroxisomes are primarily involved in the oxidative type of metabolism and have been implicated in cellular ROS homeostasis along with chloroplast and mitochondria. Abiotic stress leads to an increase in the cellular ROS concentration and if left uncontrolled it would lead to DNA damage, protein denaturation, carbohydrate oxidation, pigment breakdown, lipid peroxidation, and ultimately to cell death [88,89]. PAs have been demonstrated to promote ROS degradation by scavenging free radicles and increasing the activity of antioxidant enzymes [90,91,92]. The involvement of peroxisomes in cellular ROS homeostasis gives peroxisome localized PAO higher significance because it is the PAOs that play a crucial role in maintaining the cellular PA, which in turn modulates the cellular ROS. Hence, in the future, peroxisomal PAOs could be instrumental in designing abiotic stress-resistant and climate recalcitrant crops.

### 3.6. Peroxisomal PAO: An Evolutionary Perspective

To further understand the peroxisomal targeting signal of PAOs, an extensive bioinformatics analysis was performed. The PAO2 (AT2G43020) from the *A*. *thaliana* was used as a query sequence in the NCBI protein BLAST and various PAO orthologs were obtained. In total, 153 PAO orthologs were obtained belonging to 121 plant species. The plants belonged to monocotyledons (21 species), dicotyledons (97 species), pteridophytes (1 species), bryophytes (1 species), and green algae (1 species). In the BLAST analysis, no probable peroxisomal orthologs were obtained from diatoms and red algae; however, the representative PAO orthologs from pteridophytes, bryophytes, and green algae namely *Selaginella moellendorffi*, *Physcomitrella patens*, and *Volvox carteri*, respectively contained a canonical PTS1 (Table 1). Interestingly, one sequence belonging to the family Amborellaceae was found [93]. The family has only one genus *Amborella*, to which only one species *Amborella trichopoda* has been documented. The family Amborellaceae has neither been placed in dicots nor monocots due to the peculiar characteristics of the xylem, which has only tracheids and no vessel elements. The family Amborellaceae has been considered the sister taxon to angiosperms and phylogenetically has been placed in the most basal lineage of angiosperms [94]. The PAO from *A. trichopoda* also contains a canonical PTS1 represented by SRM>. The presence of canonical PTS1 in lower plants and *A. trichopoda* suggests that peroxisomal PAO diverges early in the evolution, which was further complemented by Salvi and Tavladoraki [95], who found that the segregation of PAO into the three domains, Eukarya, bacteria, and archaea, occurred early in the evolutionary stage.

However, not all the PAO orthologs contained canonical PTS1. In the case of monocotyledons, out of 22 sequences, 21 were found to contain canonical PTS1, while one sequence belonging to *H*. *vulgare* (KAE8772463.1) contained CRT>. The possibility of CRT being a functional PTS1 is discussed in Section 3.5. In the case of dicotyledons, out of 127 sequences, 120 contained canonical PTS1, while six sequences were found to be non-canonical. The non-canonical sequences were represented by TRL>, SRF>, and SRV> (underlined amino acids represented the non-canonical or low abundance residues). In canonical PTS1, all three residues were of high abundance. In the case of non-canonical PTS1, out of three amino acid residues, one amino acid was of low abundance while the remaining two amino acids were of high abundance. Amongst the dicotyledon, *Prunus yedoensis* belonging to the family Rosaceae contained the PAO ortholog, having IPL> as the C-terminal tripeptide, where the two residues isoleucine and proline at −3 and −2 respectively were of low abundance. The C-terminal tripeptide, IPL> has not yet been experimentally proven to be a functional PTS1. The C-terminus tripeptide combination, having two low abundance amino acid residues and one high abundance amino acid residue, is extremely rare to be a functional PTS1. However, Skoulding et al. [82] reported a C-terminus tripeptide SNV> to be a functional PTS1, where two amino acids, namely asparagine and valine at −2 and −1 positions respectively, are of low abundance in nature. Hence, the possibility of IPL> being a functional PTS1 remains positive but has yet to be tested experimentally.

**Figure 4:**
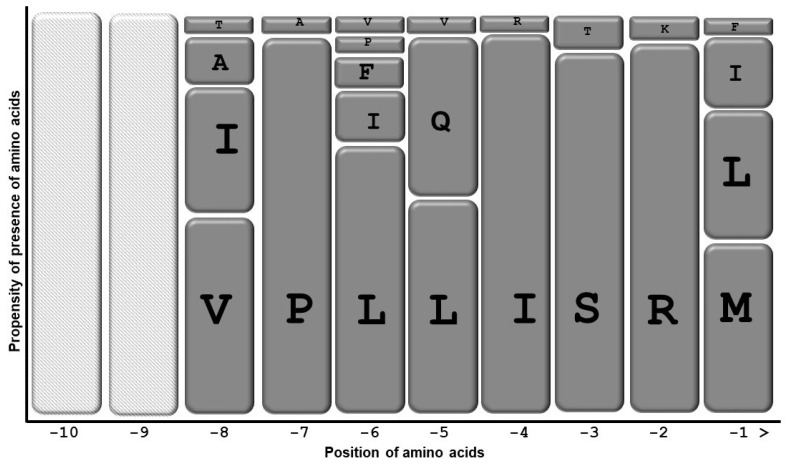
Graphical representation of PTS1 domain present in the PAO of various plants. A total of 153 PAO enzyme sequences were obtained from NCBI. The sequences were aligned using CLUSTAL W 2.1 (Supplementary Figure S1). From the multiple sequence alignments, the consensus amino acids were derived. The *y*-axis shows the propensity of the presence of specific amino acids at specific positions, while the *x*-axis represents the position of amino acids at the C-terminus of the respective protein. “>” denotes the end of the polypeptide chain. Each amino acid is represented by one rectangular box. The higher the size of the rectangle, the higher the propensity of the amino acid to remain present at that specific position. The empty rectangle represents high variability.

Amongst canonical PTS1, SRM> was found to be present in 44.7% of sequences, followed by SRL> (38.1%) and SRI> (11.1%). SKL> was found only in one scenario of green alga *Volvox carteri*. So, amongst the canonical signal in the PAOs, SRM> and SRL> were favored over SKL>. We further performed a multiple sequence alignment of C-terminal ten amino acids and deduced a probable PAO amino acid sequence. At the −1 position, methionine and leucine were favored, followed by isoleucine. Phenylalanine was found to remain present in two sequences. Phenylalanine was demonstrated to remain present at −1 positions; however, it was considered a low abundance residue at −1 [82]. At −2, −3, −4, −5, −6, −7, and −8 positions, R, S, I, L, L, P, and V predominated, respectively. At the −2 position, lysin was found to remain present at one sequence, namely *V*. *carteri* (green algae). Lysine at the −2 position was a high abundance amino acid residue that has been very often found to remain present at the −2 position in the PTS1 of higher plants constituting a PTS1 SKL>. The presence of SKL> in the green alga *V*. *carteri*, further supports the idea that bifurcation of peroxisomal PAO occurs very early in the evolutionary lineage. Figure 4 presents a diagrammatic representation of C-terminal ten amino acids of PAO enzymes from which we have deduced the probable PAO PTS1 consensus sequence to be [V/I/A/T][P/A][L/F/I/P/V][L/Q/V][I/R][S/T][R/K][M/L/I/F] at −8, −7, −6, −5, −4, −3, −2, and −1 positions, respectively. The −9 and −10 positions showed very high variability.

## 4. Conclusions

The association between polyamines (PAs), plant growth, and development has been well established. Their involvement in the abiotic stress tolerance of plants has also been observed. The PAO enzymes play a critical role in maintaining cellular concentrations of PAs. PAOs have been found to be localized in apoplast, cytosol, chloroplast, and peroxisomes. The peroxisomal localization of PAOs has been driven by PTS1. This review presents a consolidated account of peroxisomal PAOs, their expression patterns, substrate specificity, a brief summary of their signal transduction, and a probable pattern of their evolutionary origin. The spatial and temporal expression pattern of various PAO isoforms across the developmental stages of plant life suggests the specific role of various isoforms; however, in the future, mutational studies need to be conducted to confirm this. The presence of canonical PTS1 in all the plant groups, starting from green alga to monocotyledons, suggests that peroxisomal PAOs bifurcate very early in the evolution process. We also propose that apart from H_2_O_2_, Ca^2+^ and NO may be involved as probable second messengers in the PAO-mediated abiotic stress tolerance signal transduction pathway. The probable involvement of PAOs in ROS homeostasis, and their expression in response to multiple abiotic stresses, suggests they could be a potential candidate for imparting abiotic stress tolerance to plants.

## Figures and Tables

**Table 1:** The PAO2 from *A*. *thaliana* was used as a query sequence in NCBI protein BLAST and 128 dicotyledons, 22 monocotyledons, and 03 lower plants, and probable PAO sequences were obtained. The table contains the scientific names of selected plant species, their common name, family, the accession number of sequences, and the C-terminus tripeptide. Multiple sequences from the same species having the same C-terminus tripeptide have been omitted.

Sl. No.	The Scientific Name of the Plant	Common Name of the Plant	Accession No. of the Sequence	Family of Plant	C-Ter Tripeptide
Dicotyledons					
1	*Amborella trichopoda*	Amborella	>XP_006836334.1	Amborellaceae *pichon*	SRM
2	*Actinidia chinensis*	Golden kiwifruit	>PSS16430.1	Actinidiaceae	SRM
3	*Artemisia annua*	Sweet wormwood	>PWA74707.1 >PWA66356.1	Asteraceae	SRF SRM
4	*Cynara cardunculus*	Cardoon	>XP_024988081.1 >KVH97550.1	Asteraceae	SRI
5	*Lactuca sativa*	Lettuce	>XP_023736501.1	Asteraceae	SRI
6	*Helianthus annus*	Common Sunflower	>XP_021986264.1	Asteraceae	SRI
7	*Beta vulgaris*	Beet	>XP_010688837.1	Amaranthaceae	SRM
8	*Chenopodium quinoa*	Quinoa	>XP_021724622.1	Amaranthaceae	SRM
9	*Spinacia oleracea*	Spinach	>XP_021845331.1	Amaranthaceae	SRM
10	*Daucus corota*	Queen Anne’s lace	>XP_017237286.1	Apiaceae	SRI
11	*Arabis alpine*	Alpine rock cress	>KFK37143.1 >KFK35146.1	Brassicaceae	SRL SRI
12	*Arabidopsis thaliana*	Mouse ear cress	>At2g43020 >NP_191464.1	Brassicaceae	SRL SRM
13	*Arabidopsis lyrata subsp.lyrata*	Lyre-leaved rock cress	>XP_020884243.1 >XP_020880523.1	Brassicaceae	SRL SRM
14	*Brassica rapa*	Field mustard	>XP_009133564.1 >XP_009104181.1	Brassicaceae	SRL SRM
15	*Brassica napus*	Rapeseed	>XP_013687858.1 >XP_022560874.1	Brassicaceae	SRL SRM
16	*Brassica oleracea var.oleracea*	Wild cabbage	>XP_013630886.1 >XP_013588398.1	Brassicaceae	SRL SRM
17	*Capsella rubella*	Pink shepherd’s purse	>XP_006294097.1	Brassicaceae	SRL
18	*Camelina sativa*	False flax	>XP_019101215.1 >XP_010516573.1	Brassicaceae	SRL SRM
19	*Eutrema salsugineum*	Saltwater cress	>ESQ44181.1	Brassicaceae	SRM
20	*Raphanus sativus*	Radish	>XP_018485151.1 >XP_018460415.1	Brassicaceae	SRL SRM
21	*Handroanthus impetiginosus*	Pink Tumpet Tree	>PIN20378.1	Bignoniaceae	SRM
22	*Cucurbita moschata*	Crookneck pumpkin	>XP_022936044.1 >XP_022931432.1	Cucurbitaceae	SRM SRL
23	*Cucurbita maxima*	Winter Squash	>XP_022976762.1 >XP_022984772.1	Cucurbitaceae	SRL SRM
24	*Cucumis melo*	Muskmelon	>XP_008464648.1 >XP_008451845.1	Cucurbitaceae	SRL SRM
25	*Cucurbita pepo*	Field pumpkin	>XP_023535748.1 >XP_023553553.1	Cucurbitaceae	SRL SRM
26	*Momordica charantia*	Bitter Squash	>ALO20334.1	Cucurbitaceae	SPL
27	*Ipomea nil*	Blue Morning glory	>XP_019193306.1	Convolvulaceae	SRM
28	*Tarenaya hassleriana*	Spider flower	>XP_010525644.1 >XP_010550699.1	Cleomaceae	SRL SRM
29	*Carica papaya*	Papaya	>XP_021898383.1	Caricaceae	SRM
30	*Cephalotus follicularis*	Western Australian Pitcher plant	>GAV59997.1	Cephalotaceae	SRM
31	*Hevea brasiliensis*	Pará rubber tree	>XP_021665846.1	Euphorbiaceae	SRM
32	*Jatropha curcas*	Physic nut	>XP_012072709.1	Euphorbiaceae	SRM
33	*Manihot esculenta*	Cassava	>XP_021603628.1	Euphorbiaceae	SRM
34	*Ricinus communis*	Castor bean	>XP_002521588.1	Euphorbiaceae	SRM
35	*Arachis duranensis*	Wild herb	>XP_015973279.1	Fabaceae	SRL
36	*Arachis hypogea*	Peanut	>XP_025669047.1	Fabaceae	SRL
37	*Cajanus cajan*	Pigeon pea	>XP_020204978.1 >XP_020210206.1	Fabaceae	SRM SRL
38	*Cicer arietinum*	Chickpea	>XP_004491274.1 >XP_004499541.1	Fabaceae	SRF SRM
39	*Glycine max*	Soybean	>XP_003551948.1	Fabaceae	SRL
40	*Glycine soja*	Wild Soybean	>KHN12003.1	Fabaceae	SRL
41	*Lupinus augustifolius*	Blue Lupin, Narrowleaved Lupin	>XP_019455951.1	Fabaceae	SRL
42	*Mucuna pruriens*	Velvet beans	>RDX68841.1	Fabaceae	SRL
43	*Phaseolus vulgaris*	Common bean	>XP_007146297.1 >XP_007141453.1	Fabaceae	SRM SRL
44	*Medicago trancatula*	Strong-spined medlick	>XP_003617318.1 >XP_013459605.1	Fabaceae	SRI SRM
45	*Trifolium subterraneum*	Subterranean clover	>GAU12612.1 >GAU22182.1	Fabaceae	SRM SRI
46	*Trifolium pratense*	Red clover	>PNY04428.1	Fabaceae	SRI
47	*Trifolium repens*	White clover	>AQQ81875.1	Fabaceae	SRI
48	*Vigna angularis*	Adzuki bean	>XP_017436636.1 >XP_017430881.1	Fabaceae	SRM SRL
49	*Vigna radiata*	Mung bean	>XP_014490314.1 >XP_014505168.1	Fabaceae	SRM SRL
50	*Quercus suber*	Cork oak	>RLW29351.1	Fagaceae	SRM
51	*Dorcoceras hygrometricum*	---	>KZV25408.1	Gesneriaceae	SRI
52	*Juglans regia*	Common Walnut	>XP_018824097.1	Juglandaceae	SRM
53	*Genlisea aurea*	Corkscrew Plant	>EPS67202.1	Lentibulariceae	SRM
54	*Punica granatum*	Pomegranate	>OWM73258.1	Lythraceae	SRL
55	*Corchorus olitorius*	Jute mallow	>OMO68085.1	Malvaceae	SRM
56	*Corchorus capsularis*	White jute	>OMO49622.1	Malvaceae	SRM
57	*Durio zibethinus*	Duria	>XP_022739756.1	Malvaceae	SRL
58	*Gossypium Raimondi*	Cotton Plant Species	>XP_012437381.1	Malvaceae	TRL
59	*Gossypium arboretum*	Tree cotton	>XP_017642334.1	Malvaceae	TRL
60	*Gossypium hirsutum*	Upland Cotton, Mexican Cotton	>XP_016712212.1	Malvaceae	TRL
61	*Herrania umbratica*	Monkey Cacao	>XP_021274234.1	Malvaceae	SRM
62	*Theobroma cacao*	Cacao tree	>XP_007048902.2	Malvaceae	SRM
63	*Eucalyptus grandis*	Flooeded gum, rose gum	>XP_010054154.1	Myrtaceae	SRM
64	*Morus notabilis*	Mulberry Tree	>XP_024027830.1	Moraceae	SRM
65	*Nelumbo nucifera*	Water Lily	>XP_010244717.1 >XP_010275888.1	Nelumbonaceae	SRM SRL
66	*Olea europea var. sylvestris*	Olive	>XP_022871877.1	Oleaceae	SRM
67	*Sesamum indicum*	Sesame	>XP_011085441.1	Pedaliaceae	SRM
68	*Papaver somniferum*	Opium poppy	>XP_026393108.1 >XP_026391831.1	Papaveraceae	SRL SRM
69	*Macleaya cordata*	Five-Seeded Plume Poppy	>OVA19352.1	Papaveraceae	SRL
70	*Erythranthe guttata*	Seep monkeyflower	>XP_012830906.1	Phrymaceae	SRM
71	*Aquilegia caerulea*	Colorado blue columbine	>PIA45277.1 >PIA30210.1	Ranunculaceae	SRV SRL
72	*Citrus sinensis*	Sweet orange	>XP_006485009.1	Rutaceae	SRL
73	*Citrus trifoliata*	Trifoliate orange	>AJP16790.1	Rutaceae	SRL
74	*Ziziphus jujuba*	Jujube	>XP_015880626.1	Rhamnaceae	SRL
75	*Coffea canephora*	Robusta coffee	>CDP16058.1	Rubiaceae	SRM
76	*Fragaria vesca*	Wild Strawberry	>XP_004303904.1	Rosaceae	SRL
77	*Malus domestica*	Apple	>ANJ77639.1	Rosaceae	SRL
78	*Prunus yedoensis*	Yoshino cherry	>XP_011032740.1	Rosaceae	IPL
79	*Prunus persica*	Peach	>XP_007215363.2	Rosaceae	SRI
80	*Prunus avium*	Sweet cherry	>XP_021824861.1	Rosaceae	SRI
81	*Rosa chinensis*	China rose	>XP_024180697.1	Rosaceae	SRL
82	*Prunus trichocarpa*	Black cottonwood	>PNT47987.1 >XP_002306765.2	Salicaceae	SRM SRI
83	*Populus euphratica*	Desert poplar	>XP_011032740.1	Salicaceae	SRM
84	*Capsicum annuum*	Sweet and chili pepper	>XP_016541238.1	Solanaceae	SRM
85	*Capsicum baccatum*	Pepper	>PHT42735.1	Solanaceae	SRM
86	*Capsicum chinense*	Habanero type pepper	>PHU11700.1	Solanaceae	SRM
87	*Citrus clementina*	Clementine	>XP_006437065.1		SRL
88	*Nicotiana sylvestris*	Flowering Tobacco	>XP_009777198.1 >XP_009757614.1	Solanaceae	SRL SRM
89	*Nicotiana attenuata*	Coyote Tobacco	>XP_019249249.1 >XP_019262812.1	Solanaceae	SRM SRL
90	*Nicotiana tomentosiformis*	Tobacco (wild species)	>XP_009588592.1 >XP_009602218.1	Solanaceae	SRL SRM
91	*Nicotiana tabacum*	Common Tobacco	>XP_016451254.1 >XP_016478455.1	Solanaceae	SRL SRM
92	*Solanum tuberosum*	Potato	>XP_006357889.1	Solanaceae	SRM
93	*Solanum pennelli*	Wild tomato	>XP_015082560.1	Solanaceae	SRM
94	*Solanum lycopersicum*	Tomato	>XP_004243630.1	Solanaceae	SRM
95	*Populus trichocarpa*		>XP_002306765.2	Salicaceae	SRI
96	*Camelia sinensis*	Tea	>QPO25410.1 >QPO25411.1	Theaceae	SRM SRI
97	*Vitis vinifera*	Common grape vine	>XP_002282970.1	Vitaceae	SRM
Monocotyledons					
98	*Asparagus officinalis*	Sparrow grass	>XP_020270229.1	Asparagaceae	SRM
99	*Elaeis guineensis*	African Oil Palm	>XP_010909649.1	Arecaceae	SRM
100	*Phoenix dactylifera*	Date palm	>XP_008787574.1	Arecaceae	SRM
101	*Ananas comosus*	Pineapple	>XP_020102904.1	Bromeliaceae	SRI
102	*Musa acuminate*	Cavendish Banana	>XP_009399229.1	Musaceae	SRM
103	*Dendrobium catenatum*	The chained dendrobium	>XP_020682153.1	Orchidaceae	SRI
104	*Phalaenopsis equestris*	Moth orchids	XP_020579254.1	Orchidaceae	SRI
105	*Oryza sativa*	Asian Rice	>BAM17621.1	Poaceae	SRL
106	*Oryza brachyantha*	African Rice	>XP_006652847.1	Poaceae	SRL
107	*Oryza meyeriana*	South-Asian Wild Rice	>KAF0892522.1	Poaceae	SRL
108	*Triticum aestivum*	Bread Wheat	>SPT20037.1	Poaceae	SRL
109	*Aegilops tauschii*	Rough-spike hard grass	>XP_020174159.1	Poaceae	SRL
110	*Hordeum vulgare*	Barley	>KAE8785488.1 >KAE8772463.1	Poaceae	SRL CRT
111	*Brachypodium distachyon*	Purple false brome	>XP_010240449.1	Poaceae	SRL
112	*Panicum hallii*	Hall’s panicgrass	>PUZ48969.1	Poaceae	SRL
113	*Panicum miliaceum*	Proso millet	>RLM75211.1	Poaceae	SRL
114	*Zea mays*	Corn	>XP_020400822.1	Poaceae	SRL
115	*Setaria italic*	Foxtail millet	>XP_004976853.1	Poaceae	SRL
116	*Eragrostis curvula*	Lovegrass	>TVU14770.1	Poaceae	SRL
117	*Sorghum bicolor*	Great millet	>XP_002448555.1	Poaceae	SRL
118	*Dichanthelium oligosanthes*	Heller’s rosette grass	>OEL27565.1	Poaceae	SRL
Lower plants					
119	*Selaginella moellendorffi*	Spikemoss	>XP_002965599.1	Pteridophytes	SRL
120	*Physcomitrella patens*	Spreading earthmoss	>XP_001756864.1	Bryophytes	SRM
121	*Volvox carteri*	---	>XP_002954733.1	Green Algae	SKL

## Data Availability

Not applicable.

## Supplementary Materials

The following supporting information can be downloaded at: https://www.mdpi.com/article/10.3390/plants12030652/s1, Figure S1: Multiple sequence alignment of last ten amino acids of peroxisomal PAO orthologs. The AtPAO2 was used as a query in NCBI-BLASTp and 153 total probable PAO orthologs were obtained out of which 128, 22 and 03 belonged to dicotyledons, monocotyledons and lower plants respectively. The last ten amino acids were copied and used for multiple alignments. The extreme left column has the accession number followed by the name of the plant species, which is followed by D/M/LP. D—dicotyledon, M—Monocotyledons, LP—lower plant. The amino acid which are commonly present at −2, −3, −4, −5, −6, −7, −8 and −9 are highlighted with grey. At −1 leucine and methionine are present almost in equal numbers hence, at −1 leucine and methionine are highlighted with black. The uncommon amino acids are highlighted with red color. The digits at the top indicate the position of amino acids from the C-terminus end. “>” indicates the end of the polypeptide chain.
