# Supplementary material for: Plant Peroxisomal Polyamine Oxidase: A Ubiquitous Enzyme Involved in Abiotic Stress Tolerance
Source: Plants (Basel). 2023 Feb 1;12(3):652. doi: 10.3390/plants12030652 (PMC9919379; doi:10.3390/plants12030652)
Supplement: Supplementary file 1 [file plants-12-00652-s001.zip › plants-2150117-supplementary.pdf]

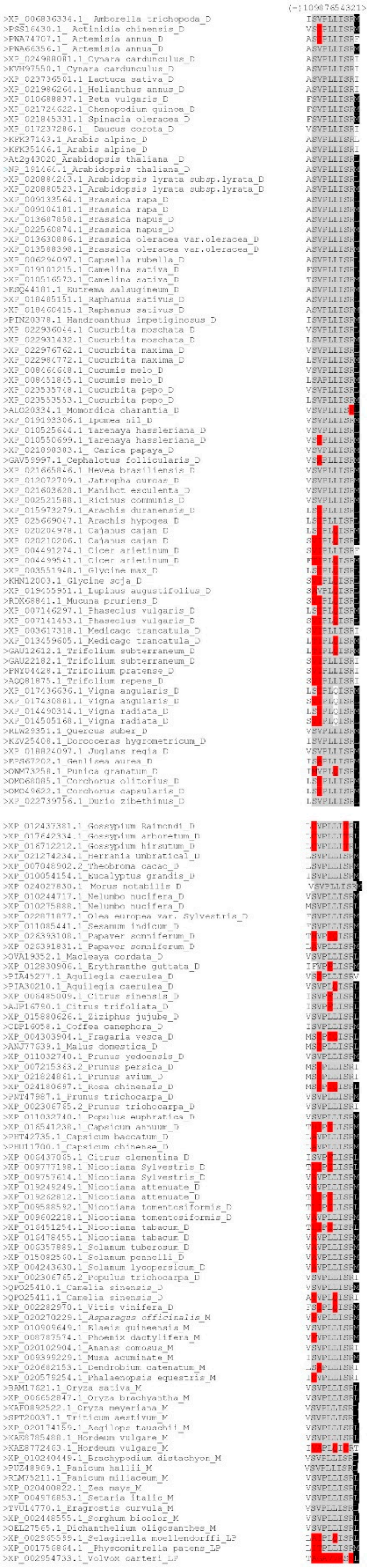

Supplementary Figure S1. Multiple sequence alignment of last ten amino acids of peroxisomal PAO orthologs. The AtPAO2 was used as a query in NCBI-BLASTp and 153 total probable PAO orthologs were obtained out of which 128, 22 and 03 belonged to dicotyledons, monocotyledons and lower plants respectively. The last ten amino acids were copied and used for multiple alignments. The extreme left column has the accession number followed by the name of the plant species, which is followed by D/M/LP. D – dicotyledon, M – Monocotyledons, LP – lower plant. The amino acid which are commonly present at -2, -3, -4, -5, -6, -7, -8 and -9 are highlighted with grey. At -1 leucine and methionine are present almost in equal numbers hence, at -1 leucine and methionine are highlighted with black. The uncommon amino acids are highlighted with red color. The digits at the top indicate the position of amino acids from the C-terminus end. “>” indicates the end of the polypeptide chain.
